# Blockade of FGF2/FGFR2 partially overcomes bone marrow mesenchymal stromal cells mediated progression of T-cell acute lymphoblastic leukaemia

**DOI:** 10.1038/s41419-022-05377-5

**Published:** 2022-11-04

**Authors:** Chen Tian, Yueyang Li, Lina Wang, Junqi Si, Yaxin Zheng, Junnan Kang, Yafei Wang, M. James You, Guoguang Zheng

**Affiliations:** 1 Department of hematology, Tianjin Medical University Cancer Institute and Hospital, National Clinical Research Center for Cancer, Key Laboratory of Cancer Prevention and Therapy, Tianjin’s Clinical Research Center for Cancer, Tianjin, 300060 China; 2 State Key Laboratory of Experimental Hematology, National Clinical Research Center for Blood Diseases, Haihe Laboratory of Cell Ecosystem, Institute of Hematology and Blood Diseases Hospital, Chinese Academy of Medical Sciences and Peking Union Medical College, Tianjin, 300020 China; 3 Department of Hematopathology, The University of Texas MD Anderson Cancer Center, Houston, TX 77479 USA

**Keywords:** Cancer microenvironment, Acute lymphocytic leukaemia

## Abstract

The development of acute lymphoblastic leuakemia (ALL) is partly attributed to the effects of bone marrow (BM) microenvironment, especially mesenchymal stromal cells (MSCs), which interact bilaterally with leukaemia cells, leading to ALL progression. In order to find MSCs-based microenvironment targeted therapeutic strategies, Notch1-induced T-cell ALL (T-ALL) mice models were used and dynamic alterations of BM-MSCs with increased cell viability during T-ALL development was observed. In T-ALL mice derived stroma-based condition, leukaemia cells showed significantly elevated growth capacity indicating that MSCs participated in leukaemic niche formation. RNA sequence results revealed that T-ALL derived MSCs secreted fibroblast growth factor 2 (FGF2), which combined with fibroblast growth factor receptor 2 (FGFR2) on leukaemia cells, resulting in activation of PI3K/AKT/mTOR signalling pathway in leukaemia cells. In vitro blocking the interaction between FGF2 and FGFR2 with BGJ398 (infigratinib), a FGFR1-3 kinase inhibitor, or knockdown FGF2 in MSCs by interference caused deactivation of PI3K/AKT/mTOR pathway and dysregulations of genes associated with cell cycle and apoptosis in ALL cells, leading to decrease of leukaemia cells. In mouse model received BGJ398, overall survival was extended and dissemination of leukaemia cells in BM, spleen, liver and peripheral blood was decreased. After subcutaneous injection of primary human T-ALL cells with MSCs, tumour growth was suppressed when FGF2/FGFR2 was interrupted. Thus, inhibition of FGF2/FGFR2 interaction appears to be a valid strategy to overcome BM-MSCs mediated progression of T-ALL, and BGJ398 could indeed improve outcomes in T-ALL, which provide theoretical basis of BGJ398 as a BM microenvironment based therapeutic strategy to control disease progression.

## Introduction

T-cell acute lymphoblastic leukaemia (T-ALL) is a heterogeneous haematological malignancy [1, 2]. Leukaemia cells destroy the normal bone marrow (BM) microenvironment, converting it to an abnormal niche [3–5] and the niche components, such as mesenchymal stromal cells (MSCs), in turn confer better survival chances to leukaemia cells, leading to the rapid progression of T-ALL [6, 7]. Therefore, it is urgent to untangle the complex interactions between leukaemia cells and MSCs and identify new therapeutic targets to treat T-ALL.

MSCs, originate from mesenchymal stem cells, provide a substantial contribution to the creation of hematopoietic niche [8]. The definition of ‘mesenchymal stem cells’ and ‘mesenchymal stromal cells’ has always been controversial. The International Society for Cell & Gene Therapy (ISCT) Mesenchymal Stromal Cell (ISCT MSC) committee issued a position paper in 2005 clarifying that the term mesenchymal stem cell was not equivalent or interchangeable with MSC. The former refered to a stem cell population with demonstrable progenitor cell function of self-renewal and differentiation, while the latter refered to a bulk population with notable secretory, immunomodulatory and homing properties [9]. And in 2019, ISCT MSC committee further consolidated and clarified functional definitions of mesenchymal stem versus stromal cells [10]. Besides, MSCs were reported to play a role in the formation of leukaemia niche. In B-ALL, MSCs were reported to be essential for the viability of in vitro cultured B lymphoblastic leukaemia cells through direct contact [11]. In AML, direct contact of leukaemia cells to MSCs could inhibit drug-induced apoptosis [12, 13]. Although MSCs-mediated protection was reported in different mechanisms [14] such as soluble cytokines, growth factors and cell interaction molecules, even establishing a link between MSC alterations and treatment outcomes, a potentially supporting role of MSCs in T-ALL has still not been adequately explored, especially no new stromal cell-based treatment strategies were developed. Therefore, our study mainly aimed to clarify the interactions between MSCs and T-ALL cells and find a new therapeutic strategy for T-ALL.

## Materials and methods

### Cell lines and reagents

T-ALL cell lines Jurkat and TALL-1 were cultured in RPMI-1640 medium supplemented with 10% fetal bovine serum (FBS). Primary MSCs and MSC cell lines (MS-5 and HS-5) were cultured in DMEM medium supplemented with 10% FBS. 293T cells were cultured in RPMI-DMEM medium with 10% FBS. RPMI-1640, RPMI-DMEM and 0.25% trypsin-EDTA were obtained from Gibco.

### Mice

The Notch1-induced murine T-ALL model was established as previously reported [14]. In order to exclude irradiation related side effects, MSCV-ICN1-IRES-GFP virus transduced BM cells of B6 mice followed by injection into non-irradiated C57 mice model were used (Fig. S1A). And normal C57 mice were used as controls. The size (Fig. S1B) and weight (Fig. S1C) of the spleen were measured. The expression of intracelluar domain of Notch1 (ICN1) in leukaemia cells was verified by realtime polymerase chain reaction (PCR; Fig. S1D) and flow cytometry was used to detect the expression of CD3 in leukaemia cells (Fig. S1E). BM smears showed that more than 90% of the cells were GFP positive (Fig. S1F) on day20 after model establishement. C57 and B6 mice were provided by Institute of Hematology and Blood Disease Hospital and all animal experiments were approved by the Animal Care and Use Committee of Tianjin Medical University Cancer Institute and Hospital and Institute of Hematology and Blood Disease Hospital.

### Multi-colour fluorescence activated cell sorting analysis

**Fig. 1:**
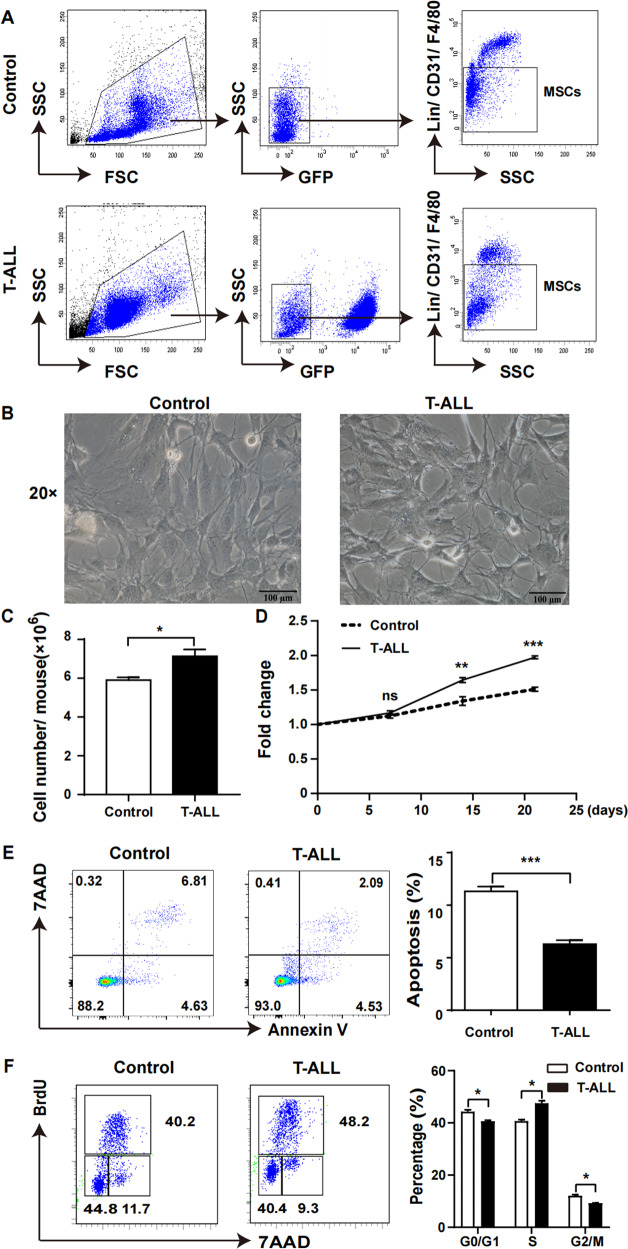
Dynamic alterations of MSCs in leukaemia niche. On day 10 after model establishment, the mice were killed and BM cells were obtained by flushing ilias, femurs and tibias. **A** FACS analysis of MSCs (GFP^-^/Lin^-^/CD45^-^/CD31^-^/F4/80^-^) from leukaemia and control mice. GFP negative selection was used to remove leukaemia cells, while CD45 and lineage were used to remove haematological cells. CD31 was used to remove endothelial cells and F4/80 was used to exclude macrophages. **B** Morphology images of MSCs isolated by FACS from leukaemia and control mice and then cultured in vitro for 14 days with DMEM. **C** The absolute number of MSCs from leukaemia mice was higher compared to control. **D** In vitro growth curve showed that the growth of leukaemia-derived MSCs was significantly faster compared to MSCs from control mice. **E** The apoptosis rate of leukaemia-derived MSCs was decreased compared to MSCs from control mice. **F** More leukaemia-derived MSCs got into S phase compared to MSCs from control mice. (*n* ≥ 3, *P < 0.05, **P < 0.01, ***P < 0.005).

**Fig. 2:**
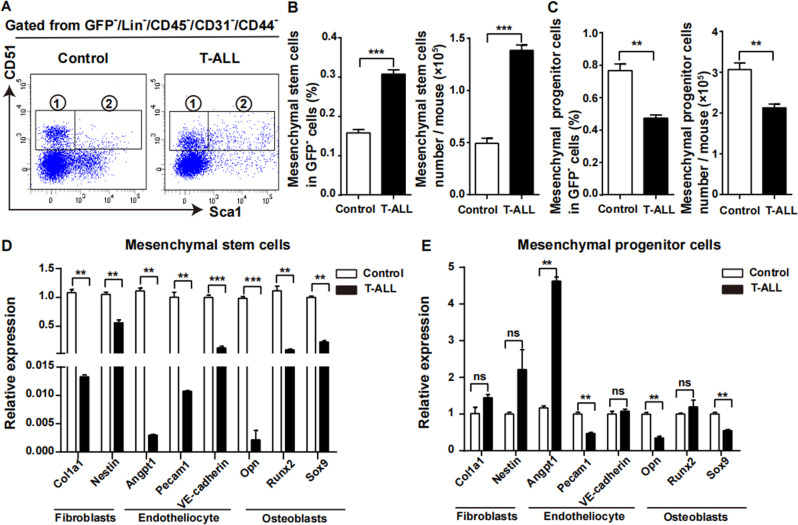
Alterations of mesenchymal stem/progenitor cells in leukaemia niche. On day 10 after model establishment, the mice were killed and mesenchymal stem and progenitor cells were analysed. **A** FACS analysis of mesenchymal stem cells (②GFP^-^CD45^-^Lin^-^CD31^-^CD44^-^CD51^+^SCA1^+^) and mesenchymal progenitor cells (①GFP^-^CD45^-^Lin^-^CD31^-^CD44^-^CD51^+^Sca1^-^). **B** The proportion and absolute number of mesenchymal stem cells from leukaemia mice were higher than that of control mice. **C** The proportion and absolute number of mesenchymal progenitor cells from leukaemia mice were lower than that of control mice. **D** Realtime PCR results revealed that the expression of mesenchymal lineage-associated genes in mesenchymal stem cells from leukaemia mice were down-regulated compared to control mice. **E** The expression of most mesenchymal lineage-associated genes in mesenchymal progenitor cells showed no significant change between leukaemia and control mice. (*n* ≥ 3, **P < 0.01, ***P < 0.005).

BM cells were obtained by flushing ilias, femurs and tibias. Mononuclear cells were separated from BM by Ficoll and then stained with CD45 MicroBeads (Miltenyi Biotec). CD45 negative cells after magnetic bead sorting (MACS) were stained with hematopoietic lineage markers Ter119, B220, CD19, CD3, Gr1, CD11b to further remove hematopoietic cells. CD31, CD44, CD51, F4/80 and Sca1 were used to sub-fractionate into three cell types: GFP^-^CD45^-^Lin^-^CD31^-^F4/80^-^ MSCs (Fig. 1A), GFP^-^CD45^-^Lin^-^CD31^-^CD44^-^CD51^+^Sca1^-^ mesenchymal progenitor cells and GFP^-^CD45^-^Lin^-^CD31^-^CD44^-^CD51^+^Sca1^+^ mesenchymal stem cells (Fig. 2A). All antibodies were listed in Table S1. The sorting markers used for MSCs, mesenchymal progenitor and stem cells were referenced [15].

### Calculation formula of absolute cell value

Total cell number of mononuclear cells in BM was calculated. The calculation formula of absolute number of MSCs = [MSCs (%)/(1-GFP^+^ (%)] × total BM mononuclear cells. The calculation formula of absolute number of mesenchymal stem/progenitor cells = [mesenchymal stem/ progenitor cells (%)/(1-GFP^+^ (%))] × total BM mononuclear cells.

### BrdU incorporation and apoptosis analysis

To test the cell cycle of MSCs in vivo, each mouse was intraperitoneally injected 150 μL APC-BrdU (BD Biosciences) 16 h before killing. To test the cell cycle of T-ALL cells co-cultured with MSCs in vitro, APC-BrdU was added and incubated for 6 h and then labled with anti-BrdU conjugated antibody and analysed by fluorescence activated cell sorting (FACS; Becton Dickinson Aria II).

Cells were stained with Annexin-V and 7-AAD (BD Pharmingen) for 20 min at room temperature. The rate of apoptosis was assessed by FACS.

### Realtime polymerase chain reaction

RNA was isolated using a Qiagen RNeasy mini kit and then synthesised to cDNA using SuperScript III (Invitrogen). The TB Green Premix Ex Taq was obtained from TaKaRa Biotech. Real-time PCR was performed using ABI-Prism 7900 sequence detection (Applied Biosystems). The expression level of target genes was analysed based on the relative quantity (RQ) value, which was calculated using the △△Ct method. All primer sequences were listed in Table S2.

### Co-culture system and drug treatment

MSCs from leukaemia and control mice were isolated by MACS and FACS with GFP^-^CD45^-^Lin^-^CD31^-^F4/80^-^ markers and then cultured in a six-well plate with DMEM. When the confluence reached 90%, T-ALL cells were added at the proportion of 3:1. The FGFR inhibitor BGJ398 was added at 1 μM (Selleckchem).

### RNA-seq analysis

MSCs were sorted from BM of T-ALL and control mice and two mice were used per group. Total RNA extracted from primary murine MSCs was sent to ShangHai Majorbio company (www.majorbio.com) for RNA sequence analysis. RNA was reverse transcribed into cDNA and hybridised with specific sequences to form miRNA/cDNA hybridisation. The fluorescent labelling on the RNA probe was separated by ribonuclease, and then the initial fluorescence intensity and the fluorescence intensity after enzyme digestion were compared to obtain the degradation amount of the RNA probe, thereby obtaining the expression of miRNA. The RNA expression profiles of MSCs from T-ALL and control mice were compared as Fig. 4A. Differentially expressed (fold change>2) RNAs with an adjusted cutoff *P* value of 0.01 of MSCs from different niche were selected through the analysis system on majorbio website (Table S3). Leukaemia cells derived from T-ALL mice were co-cultured with T-ALL or control mice derived MSCs in vitro for 16 hours and then leukaemia cells were sorted for RNA-seq. Figure 4B showed the heat maps of leukaemia cells co-cultured with MSCs from different origins. Total transcript levels of primary murine BM-MSCs from different groups and primary murine T-ALL cells co-cultured with primary murine MSCs from different origins were shown in Tables S4 and S5, respectively, which were obtained from the analysis system on majorbio website. Enrichment analysis of functions and signalling pathways of the target genes was conducted by Gene Ontology (GO) database (Fig. S2), which could divide the functions of genes into three parts: cellular component (CC), molecular function (MF) and biological process (BP).

### Western blot analysis

Proteins were extracted and then analysed by SDS-PAGE. Immunoblotting was performed using antibodies against PI3K (AB191606, Abcam), AKT1 + AKT2 + AKT3 (AB179463, Abcam), phospho-AKT (AB192623, Abcam), mTOR (AB32028, Abcam), phospho-mTOR (AB109268, Abcam), phospho-PI3K p85 (AF3241, Affinity Biosciences), p21 (AB109520, Abcam), p27 (AB32034, Abcam), BAX (AB32503, Abcam) and CDK2 (AB32147, Abcam).

### Lentivirus-based knockdown of FGF2

The lentivirus vector pLVX-FGF2-mcherry and pLVX-mcherry scramble were obtained from Youbio Biosciences Inc. The single-strand DNA oligos were annealed to form a double-strand oligos and then ligated to the pLVX-shRNA-mcherry lentivirus to construct shRNA vectors. Figure S3A showed a schematic diagram of the vector for intuitive elaboration.

The lentivirus vector plasmids (1.2 μg) combined with packaging plasmids pSPAX_2_ (0.6 μg) and pMD2.G (0.6 μg) were co-transfected into 293T cells to produce virus, using Lipofectamine 2000 (Life Technologies). Fluorescence microscope images of 293T cells packing virus were shown as Fig. S3B. Forty-eight hours later, the supernatants were collected and transfected MSCs. Fluorescence image (Fig. S3C) and FACS (Fig. S3D) revealed that the efficiency of viral infection was high. Realtime PCR results revealed that FGF2-shRNA could effectively knockdown the expression of FGF2 (Fig. 4G).

### Subcutaneous tumour formation assay

NOD/SCID mice were inoculated subcutaneously with 1 × 10^7^ primary human T-ALL cells no matter there was Notch mutation or not, suspended in 100 μl phosphate-buffered saline (PBS) with HS-5 at a ratio of 3:1. FGFR inhibitor BGJ398 was intraperitoneal injected every 2 days for a total of seven times (30 mg/kg, 25 µl/mouse). The control mice were treated intraperitoneally with PBS. T-ALL patient samples were obtained from Tianjin Medical University Cancer Institute and Hospital. This study was subject to approval by the Research Ethics Committee of Tianjin Medical University Cancer Institute and Hospital. All methods were carried out in accordance with relevant guidelines and regulations. Informed consent was obtained from all patients.

HS-5 transfected with scramble or shRNA-FGF2 were injected subcutaneously into NOD/SCID mice with 1 × 10^7^ primary human T-ALL cells, and the size of the resulting tumours was measured using a caliper on day 30 after injection.

### Interruption of FGF2 and FGFR2 in mouse model

NOD/SCID mice were injected by tail vein with 1 × 10^6^ primary murine T-ALL cells with MS-5 and then intraperitoneal injected FGFR inhibitor BGJ398 (30 mg/kg/200 µL) since day7 after model establishment, every 2 days for a total of four times. Nine NOD/SCID mice were used for each group. On day14 after transplantation, three mice were killed and the proportion of T-ALL cells in peripheral blood (PB) and BM was determined, the spleen and liver were weighed and the residual normal hematopoietic stem and progenitor cells were tested. The residual six mice per group were used for survival analysis, with the criteria to determine the death of mice that the mice stopped breathing and heartbeat, and did not respond to external stimuli. All mice were identified to be died of leukaemia with flow cytometry validation.

### Statistical analysis

Data were summarised as means±standard deviations (SD). The significance of differences was assessed using the Student’s *t* test. *P* values <0.05 were considered significant. Kaplan–Meier method was used to generate survival curve. Statistical analyses were performed using Graphpad Prism software 8.0.

## Results

### Dynamic alterations of MSCs under leukaemic microenvironment

On day 10 after mice model establishment, the mice were killed and BM-MSCs (GFP^-^CD45^-^Lin^-^CD31^-^F4/80^-^) were isolated by MACS and FACS (Fig. 1A). Due to the infiltration of leukaemia cells in BM, the percentage of MSCs couldn’t accurately reveal the reality. Therefore absolute number of MSCs was used to calculate and our results showed that absolute number of MSCs from T-ALL mice was higher than that from control mice (Fig. 1C). However, no difference in the morphology of MSCs from these two groups was observed (Fig. 1B).

In vitro culture of MSCs revealed that the growth rate of leukaemia-derived MSCs was substantially elevated (Fig. 1D). Cell cycle and apoptosis analysis demonstrated that more leukaemia-derived MSCs entered into S phase (Fig. 1F) with decreased apoptosis (Fig. 1E), indicating that leukaemic cells altered the growth capacity of MSCs in the leukaemic microenvironment.

### Changes of mesenchymal stem and progenitor cells in leukaemic niche

MSCs are originated from mesenchymal stem and progenitor cells, which demonstrate capacity of self-renewal and differentiation to fibroblasts, endotheliocytes and osteoblasts. Our results revealed that on day 10 after model establishment, the percentage of mesenchymal stem cells in GFP negative (GFP^-^) cells and their absolute number from leukaemia mice were higher than that of control mice (Fig. 2B), while the situation was opposite for mesenchymal progenitor cells (Fig. 2C).

Real-time PCR results showed that the characteristic genes associated with mesenchymal lineage differentiation in leukaemia-derived mesenchymal stem cells were decreased (Fig. 2D), indicating that the angiogenic, adipogenic and osteogenic differentiation potential of mesenchymal stem cells from leukaemia mice were decreased. However the expression of much of these genes in mesenchymal progenitor cells showed no significant difference between these two groups (Fig. 2E). These results indicated that mesenchymal stem cells did not differentiate downward, while the differentiation ability of mesenchymal progenitor cells was not changed, resulting in the number of mesenchymal stem cells increased and mesenchymal progenitor cells was exhausted.

### MSCs accelerated the proliferation of T-ALL cells

**Fig. 3:**
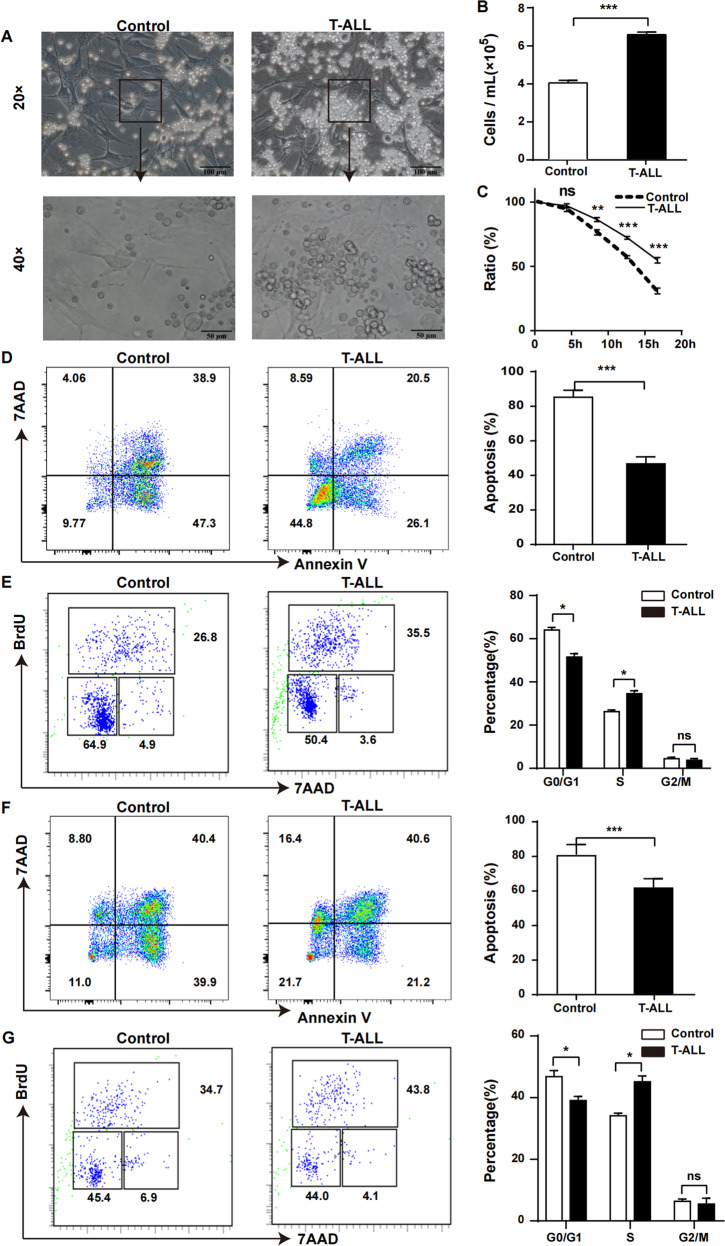
MSCs promoted growth of leukaemia cells. MSCs isolated from leukaemia and control mice by FACS were cultured in DMEM. When the confluence reached 90%, T-ALL cells were added at the proportion of 3:1. **A** Morphology of primary murine T-ALL cells after co-culture with MSCs for 16 h (direct contact). **B** After co-culture for 16 hours, the absolute number of leukaemia cells was counted and results revealed that the absolute number of leukaemia cells was higher in leukaemia-derived MSCs group compared to control. **C** Growth curve showed that the viability of T-ALL cells co-cultured with MSCs from leukaemia was better compared to control. Vertical legend meant the ratio of surviving leukaemia cells to the initial number of leukaemia cells. **D** Apoptosis rate of T-ALL cells co-cultured with MSCs from leukaemia mice decreased compared to control mice. **E** BrdU analysis revealed that more T-ALL cells co-cultured with MSCs from leukaemia mice got into S phase compared to control mice. **F**, **G** T-ALL cells were co-cultured with MSCs from leukaemia and control mice in indirect contact using transwell chamber. Apoptosis and BrdU analysis showed the same trend as direct contact. (*n* ≥ 3, *P < 0.05, **P < 0.01, ***P < 0.005).

To further determine the contribution of MSCs during T-ALL development, an in vitro co-culture system was established. Morphological images intuitively revealed that the number of surviving leukaemia cells after co-culture with leukaemia-derived MSCs for 16 h was higher compared to control group (Fig. 3A) and the absolute number of T-ALL cells in leukaemia MSCs group was higher than control group (Fig. 3B). The growth curve also showed the same trend (Fig. 3C) suggesting that MSCs contributed to the survival of leukaemia cells. Apoptosis rate of leukaemia cells co-cultured with leukaemia-derived MSCs for 16 h was significantly decreased (Fig. 3D), whereas the ratio of S phase cells was significantly increased (Fig. 3E), indicating that the proliferation of leukaemia cells was remarkably modified by leukaemia-derived MSCs. The apoptosis rate and survival data of T-ALL cells cultured alone in vitro for 16 h were shown in Fig. S4A, B respectively.

To better understand how MSCs interacted with leukaemia cells, a transwell indirect contact co-culture experiment was conducted. Even though MSCs and leukaemia cells were not in direct contact, the apoptosis rate of leukaemia cells was still decreased (Fig. 3F), and the S phase proportion was significantly elevated (Fig. 3G), demonstrating that MSCs could affect the growth of leukaemia cells in an indirect contact manner.

### FGF2/FGFR2 combination activated PI3K/AKT/mTOR pathway in leukaemia cells

**Fig. 4:**
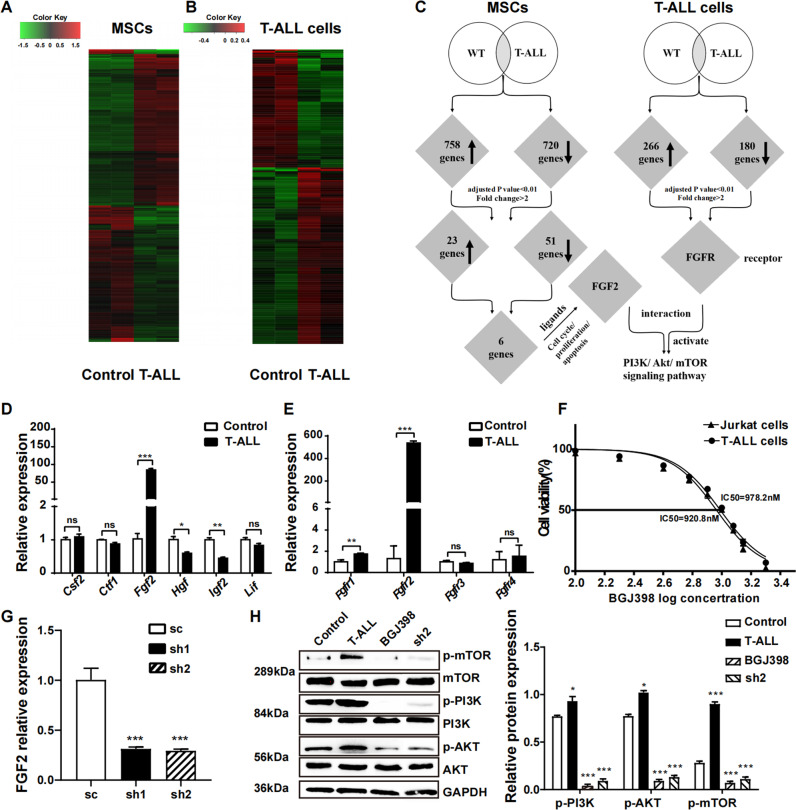
FGF2 secreted by MSCs bound to FGFR2 on T-ALL cells and activated PI3K signalling pathway. **A** RNA-seq cluster diagram of primary murine MSCs isolated from leukaemia and control mice (Red colour meant the genes were up-regulated and green colour meant the genes were down-regulated). **B** Heat map of primary murine T-ALL cells co-cultured with MSCs from leukaemia or control mice for 16 h (red colour meant the genes were up-regulated and green colour meant the genes were down-regulated). **C** Schematic representation of analysis strategies integrating expression profiles obtained from two RNA-seqs. **D** Realtime PCR was used to validate the expression pattern of selected six cytokine genes in MSCs and found that FGF2 was significantly up-regulated in MSCs from leukaemia mice. **E** Quantitative analysis of four FGF receptors showed that FGFR2 was significantly up-regulated in murine T-ALL cells cultured with leukaemia derived MSCs. **F** Dose response curve of BGJ398 with different concentration revealed that IC50 was 1 μM. **G** The knockdown efficiency of FGF2 in MSCs after sh-RNA transfection. **H** The phosphorylation of key proteins of PI3K/AKT/mTOR signalling pathway in T-ALL cells after addition of BGJ398 or FGF2 knockdown were analysed by Western blot. (*n* ≥ 3, *P < 0.05, **P < 0.01, ***P < 0.005).

Heat map difference of primary murine MSCs between T-ALL and control mice were shown as Fig. 4A and 1478 genes with 758 up-regulated genes and 720 down-regulated genes were found to be differentially expressed between MSCs from T-ALL and control mice (Fig. 4C). Seventy-four genes, expressed significantly differently in leukaemia mice derived MSCs compared to control (fold change>2, adjusted *P* value <0.01), were selected for further analysis, including 23 genes up-regulated and 51 genes down-regulated (Table S3). After GO analysis of the seventy-four genes, six secreted cytokines related to cell cycle, proliferation and apoptosis including *CSF2*, *CTF1*, *FGF2*, *IGF2*, *HGF* and *LIF* were selected for PCR verification and *FGF2* was confirmed to be significantly up-regulated in leukaemia mice derived MSCs (Fig. 4D).

RNA-seq of primary murine T-ALL cells co-cultured in vitro with MSCs derived from T-ALL or control mice for 16 h were conducted (Fig. 4B) and the results revealed 446 differentially expressed genes, including 266 genes up-regulated and 180 genes down-regulated (Fig. 4C). *FGFR1 and 2*, the receptor of *FGF2*, were differently expressed in leukaemia cells co-cultured with MSCs from different origin and *FGFR2* expression was significantly increased in leukaemia cells co-cultured with MSCs from T-ALL mice after PCR validation (Fig. 4E).

To verify the interaction between FGF2 and FGFR2 to the progression of T-ALL, FGF2-shRNA vector was constructed and the knockdown efficiency in MSCs was high (Fig. 4G). The dose-response curves of BGJ398 (Infigratinib), an oral FGFR1-3 selected tyrosine kinase inhibitor (TKI), revealed that IC50 was 1 μM for in vitro experiments (Fig. 4F).

Kyoto Encyclopedia of Genes and Genomes (KEGG) and Cell Signal Technology (CST) database both revealed that *FGF2/FGFR2* interaction could activate PI3K/AKT/mTOR pathway. In our study, GO analysis of leukaemia cells revealed that PI3K/AKT pathway was activated in leukaemia cells co-cultured with T-ALL derived MSCs (Fig. S2B) and the protein expression level of p-PI3K, p-AKT, p-mTOR in leukaemia cells were also increased after co-culture with T-ALL derived MSCs. However, after addition of BGJ398 or FGF2 knockdown, the expression of phosphorylation of PI3K/AKT/mTOR proteins were significantly reduced (Fig. 4H), indicating that FGF2/FGFR2 combination activated PI3K/AKT/mTOR pathway in leukaemia cells.

### Blockade of FGF2 and FGFR2 suppressed the growth of T-ALL cells in vitro

**Fig. 5:**
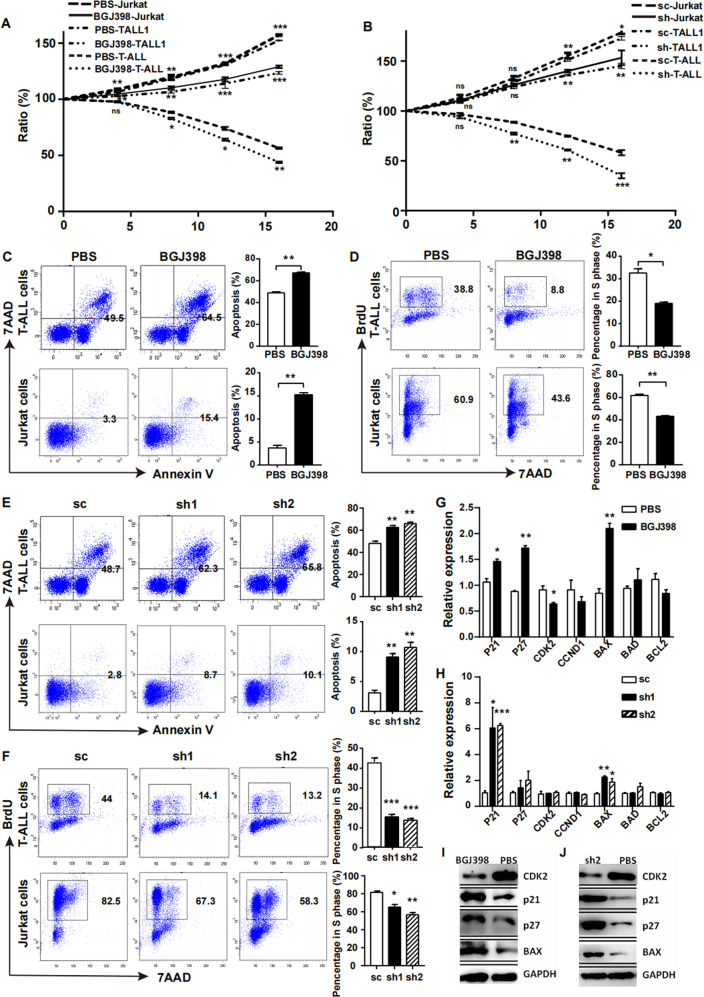
The growth of T-ALL cells was suppressed after FGF2/FGFR2 blockade in vitro co-culture system. **A** Growth curve of Jurkat, TALL-1 and primary murine T-ALL cells co-cultured with MSCs from leukaemic mice supplemented with BGJ398. Vertical legend meant the ratio of surviving leukaemia cells to the initial number of leukaemia cells. **B** Jurkat, TALL-1, or primary murine T-ALL cells were co-cultured with leukaemic mice derived MSCs. Growth curve showed that the growth of T-ALL cells were inhibited after FGF2 knockdown in MSCs. **C** BGJ398 (1 μM) was added to the co-culture system and 12 h later, the apoptosis rates of primary murine T-ALL cells (upper), Jurkat (lower) were increased. **D** Percentage of T-ALL cells in S phase was decreased after 12 h co-culture with BGJ398. **E** The apoptosis rates of primary murine T-ALL cells (upper), Jurkat (lower) were increased after co-culture with FGF2 knockdown MSCs for 12 h. **F** Percentage of T-ALL cells in S phase was decreased after co-culture with FGF2 knockdown MSCs for 12 h. **G** PCR results revealed that mRNA expression of cell cycle and apoptosis associated genes in T-ALL cells changed after addition of BGJ398 to the co-culture system. **H** Expression changes of cell cycle and apoptosis associated mRNA in T-ALL cells after co-culture with FGF2 knockdown MSCs. **I**, **J** Western blot results also revealed that P21, P27 and BAX were up-regulated and CDK2 was down-regulated after FGF2 and FGFR2 blockade. (*n* ≥ 3, *P < 0.05, **P < 0.01, ***P < 0.005).

Two ways were used to block the interaction between FGF2 and FGFR2: one way used shRNA to knockdown FGF2 in MSCs, the other way was FGFR inhibitor BGJ398 to competitively combined with FGFR2 in leukaemia cells. After 16 hours, both of the growth of primary murine T-ALL cells, and T-ALL cell lines Jurkat and TALL-1 were inhibited (Fig. 5A), with increased apoptosis (Fig. 5C) and decreased S phase ratio (Fig. 5D). The same situation was observed in T-ALL cells co-cultured with FGF2-shRNA transfected MSCs (Fig. 5B, E, F), no matter primary murine T-ALL cells, or T-ALL cell line Jurkat were used. Realtime PCR results revealed that the expression of cell cycle and apoptosis associated genes, such as *P21, P27, CCND1, CDK2, BAX, BAD* and *BCL2* were altered in primary T-ALL cells after FGF2 and FGFR2 blockade (Fig. 5G, H). Cell cycle inhibition gene *P21/P27* and pro-apoptosis gene *BAX* were increased in primary T-ALL cells after FGF2 and FGFR2 blockade, while cell cycle promoting gene *CDK2* was decreased. The same results were repeated in Jurkat (data not shown). Western blot results also revealed that P21, P27 and BAX were up-regulated and CDK2 was down-regulated after FGF2 and FGFR2 blockade (Fig. 5I, J).

We tested the proliferation and apoptosis of MSCs after FGF2 knockdown (Fig. S5A, B) and found that the growth capacity of MSCs was reduced (Fig. S5C). However, after addition of BGJ398, there’s no effect to the growth of MSCs (data not shown). These results revealed that FGF2 knockdown could attenuate the growth of MSCs that could explain why they can’t support leukaemic cell proliferation/survival.

### Blockade of FGF2 and FGFR2 prolonged T-ALL mice survival

**Fig. 6:**
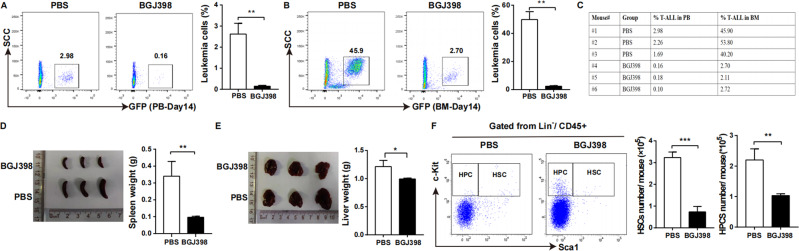
The progression of T-ALL was significantly delayed after FGF2/FGFR2 blockade in vivo. **A** The engraftments of T-ALL cells in PB were significantly decreased on day14 after model establishment when the mice were injected intraperitoneally with BGJ398. **B** The engraftments of T-ALL cells in BM were similarly prominently decreased on day14 after model establishment when FGF2 and FGFR2 combination was disrupted. **C** The dynamic monitoring of the proportion of T-ALL cells in PB and BM were summarised. **D** The size and weight of spleen from FGF2/FGFR2 blockade mice were reduced on day14 after model establishment. **E** The size and weight of liver from FGF2/FGFR2 blockade mice was shrunk on day14 after model establishment. **F** To intuitively understand the changes of the number of normal HSCs and HPCs in BM of T-ALL after FGF2/FGFR2 blockade, FACS was used and results showed that both number of them were decreased. (*n* ≥ 3, *P < 0.05, **P < 0.01, ***P < 0.005).

**Fig. 7:**
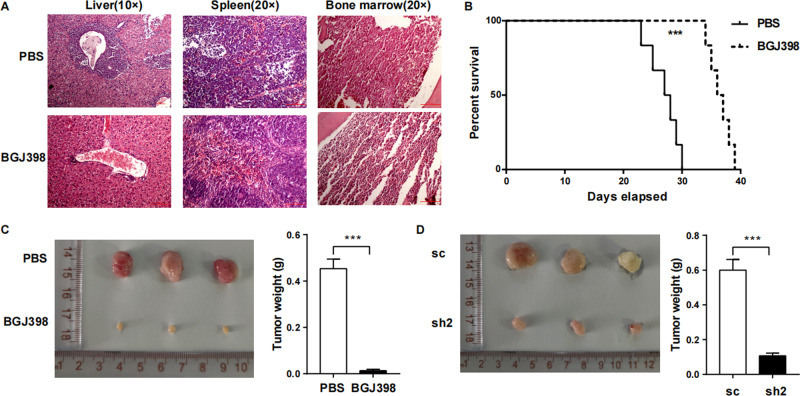
The survival of T-ALL was significantly prolonged after FGF2/FGFR2 blockade in vivo. **A** On day 14 after model establishment, the mice were killed. Biopsy of liver, spleen and BM of leukaemia mice stained with hematoxylin-eosin (H & E) revealed that infiltration of leukaemia cells to these organs were decreased after BGJ398 treatment. **B** The survival curve indicated that prolonged survival was observed in FGF2/FGFR2 blockade mice. (*n* = 6) **C** Subcutaneous tumour formation assay revealed that the tumour size and weight in BGJ398 group on day 30 after model establishment were smaller compared to PBS group. **D** Knockdown of FGF2 in HS-5 also significantly inhibited subcutaneous tumour growth compared to control group on day 30 after model establishment. (*n* = 3, ***P < 0.005).

On day14 after model establishment, mice were killed and FACS analysis indicated much lower engraftment of T-ALL cells in PB (Fig. 6A) and BM (Fig. 6B) of BGJ398 recipients compared to PBS recipients. The engraftment ratios in PB and BM were summarised in Fig. 6C. Disease deceleration was manifested by slower development of splenomegaly (Fig. 6D) and hepatomegaly (Fig. 6E), and less T-ALL cell infiltration in the spleen, liver and BM (Fig. 7A) of mice receiving BGJ398 compared to mice receiving PBS. Interestingly, we observed a longer latency of symptomatic T-ALL and prolonged overall survival time in BGJ398 mice compared with control mice (Fig. 7B).

Our previous results found that normal hematopoietic stem cells (HSCs) in leukaemic mice were kept in a more quiescent state with increased number while normal hematopoietic progenitor cells (HPCs) demonstrated accelerated proliferation and exhaustion [4, 14]. To intuitively understand the number changes of HSCs and HPCs after FGF2 and FGFR2 blockade, we detected HSCs (CD45^+^GFP^-^Lin^-^Sca1^+^c-Kit^+^) and HPCs (CD45^+^GFP^-^Lin^-^Sca1^-^c-kit^+^) in BM by FACS and results revealed that both of them were decreased (Fig. 6F).

To validate the effect of FGF2-FGFR2 interaction to human T-ALL, two methods to block FGF2 and FGFR2 were used for subcutaneous assay. As shown in Fig. 7C, primary human T-ALL cells with HS-5 were injected subcutaneously and BGJ398 or PBS was intraperitoneal injected every 2 days for a total of seven times, the growth of tumours in mice with FGF2 and FGFR2 blockade was slower than control mice (*p* < 0.05). For Fig. 7D, HS-5 transfected with scramble or shRNA-FGF2 were injected subcutaneously into NOD/SCID mice with primary human T-ALL cells, and the size of tumours in shRNA-FGF2 group was smaller compared to scramble group.

## Discussion

BM microenvironment provides supporting signals and protective clues for the proliferation, survival and adhesion of leukaemia cells, leading to the initiation and progression of leukaemia [16–19]. MSCs were reported as a protector in the drug resistance of T-ALL cells. Wang et al found that Jurkat cells adhered to MSCs in the co-culture system, which was mediated by ICAM-1. Treatment against ICAM-1 led to decreased mitochondria transfer and increased chemotherapy-induced cell death [20]. Cai et al. [21] also found that MSCs could trigger Drp1 activation-induced changes in mitochondrial dynamics, which was crucial to protect T-ALL cells against chemotherapeutic agents. However, the contributions of BM-MSCs to T-ALL leukaemia niche formation and progression remain poorly defined. Thus, there is great need to understand the role of BM-MSCs in T-ALL in order to identify therapeutic targets in the niche. Our study demonstrated that T-ALL BM niche derived MSCs had abnormal expansion viability without morphology alteration. BM-MSCs in turn promoted the growth of T-ALL cells through FGF2 and FGFR2 interaction which activated PI3K/AKT/mTOR pathway of T-ALL cells. Blockade the combination between FGF2 and FGFR2 remarkably decreased the infiltration of T-ALL cells in BM, PB, liver and spleen and prolonged the survival of T-ALL mice.

We found that the BM-MSCs in leukaemic microenvironment had significantly enhanced ability of proliferation and reduced apoptosis. However, BM-MSCs are not equivalent or interchangeable with mesenchymal stem cells. The ISCT MSC committee announces that mesenchymal stem cells refer to a stem cell population with progenitor cell functionality, whereas MSCs refer to a bulk population with notable secretory, immunomodulatory and homing properties [10]. As to the gating strategy, we refered to some high-level papers focused on MSC research [15, 22–24]. Our study showed that the absolute number of mesenchymal stem cells was higher in leukaemia mice than control mice while that of mesenchymal progenitor cells were decreased. The differentiation associated genes to diverse directions such as osteogenesis, endotheliogenesis and fibrogenesis were down-regulated in mesenchymal stem cells, suggesting that their differentiation ability to these directions were decreased, which resulted in the increase of their numbers in BM. However, the differentiation genes in mesenchymal progenitor cells showed no difference, resulting in the decrease of their numbers in BM.

The leukaemic BM niche contributed to the massive expansion of leukaemic cells. Our results revealed that after co-culture with T-ALL derived MSCs, the proliferation of T-ALL cells were accelerated, suggesting that MSCs in turn promoted the malignant amplification of T-ALL cells.

FGFs comprise a large family of growth factors that regulate cell survival, growth and differentiation [25–29]. FGFs bind to four FGFRs, triggering several signal pathways, including PI3K/Akt/mTOR pathway [30–34]. Recently, increasing evidence showed that FGF2 was important in the pathogenesis of haematological malignant tumours [34–38], such as chronic myeloid leukaemia (CML), chronic lymphoid leukaemia (CLL) and Hodgkin’s lymphoma. However, the role of FGF2 in T-ALL is not demonstrated. Our data demonstrated in T-ALL microenvironment, MSCs secreted amounts of FGF2, which bind to FGFR2 on T-ALL cells, leading to the activation of PI3K/Akt/mTOR pathway of T-ALL cells.

Infigratinib, also named BGJ-398, is an oral FGFR1-3 selective tyrosine kinase inhibitor (TKI) which was approved for adults with previously treated, unresectable locally advanced or metastatic cholangiocarcinoma on 28 May 2021 by the Food and Drug Administration (FDA). BGJ398 is in clinical trials for the treatment of squamous cell non-small-cell lung cancer, gastrointestinal stromal tumour [39, 40]. Several papers reported that FGFR1 activation was associated with stem cell leukaemia/lymphoma syndrome (SCLL) and BGJ398 could be used in the treatment of SCLL [41–43]. However, the effect of BGJ398 in leukaemia especially T-ALL was rarely known. We found that knockdown FGF2 expression by sh-RNA in MSCs or addition of BGJ398 could deactivate PI3K/Akt/mTOR pathway of T-ALL cells with up-regulation of *P21*, *P27*, *BAX* and down-regulation of *CDK2*, resulting in growth suppression of T-ALL cells in vitro. For in vivo experiment to verify FGF2-FGFR2 interaction, we used two methods: one was caudal vein injection model, the other was subcutaneous tumour formation assay. The former was injected by tail vein with primary Notch1 induced murine T-ALL cells and the latter was inoculated subcutaneously with primary human T-ALL cells from T-ALL patients no matter there was Notch mutation or not. Our results revealed that the role of FGF2/FGFR2 crosstalk was not specific for Notch1 overexpression induced T-ALL model, but also could be of general relevance for T-ALL. However, more experiments were needed to verify this.

## Conclusion

In all, our findings clarified that FGF2 and FGFR2 interaction mediated the proliferative activity of MSCs to T-ALL cells. Genetic targeting FGF2 in MSCs or FGFR2 antagonism BGJ398 led to suppressed T-ALL progression.

## Supplementary information

original data files

original data files

original data files

original data files

supplemental figure S1

supplemental figure S2

supplemental figure S3

supplemental figure S4

supplemental figure S5

supplemental figure legend

aj-checklist

supplemental table 1

supplemental table 2

supplemental table 3

supplemental table 4

supplemental table 5

## Data Availability

The data sets used and/or analysed during the current study are available from the corresponding author on reasonable request.
