# Supplementary figures and images for: Blockade of FGF2/FGFR2 partially overcomes bone marrow mesenchymal stromal cells mediated progression of T-cell acute lymphoblastic leukaemia
Source: Cell Death Dis. 2022 Nov 4;13(11):922. doi: 10.1038/s41419-022-05377-5 (PMC9636388; doi:10.1038/s41419-022-05377-5)

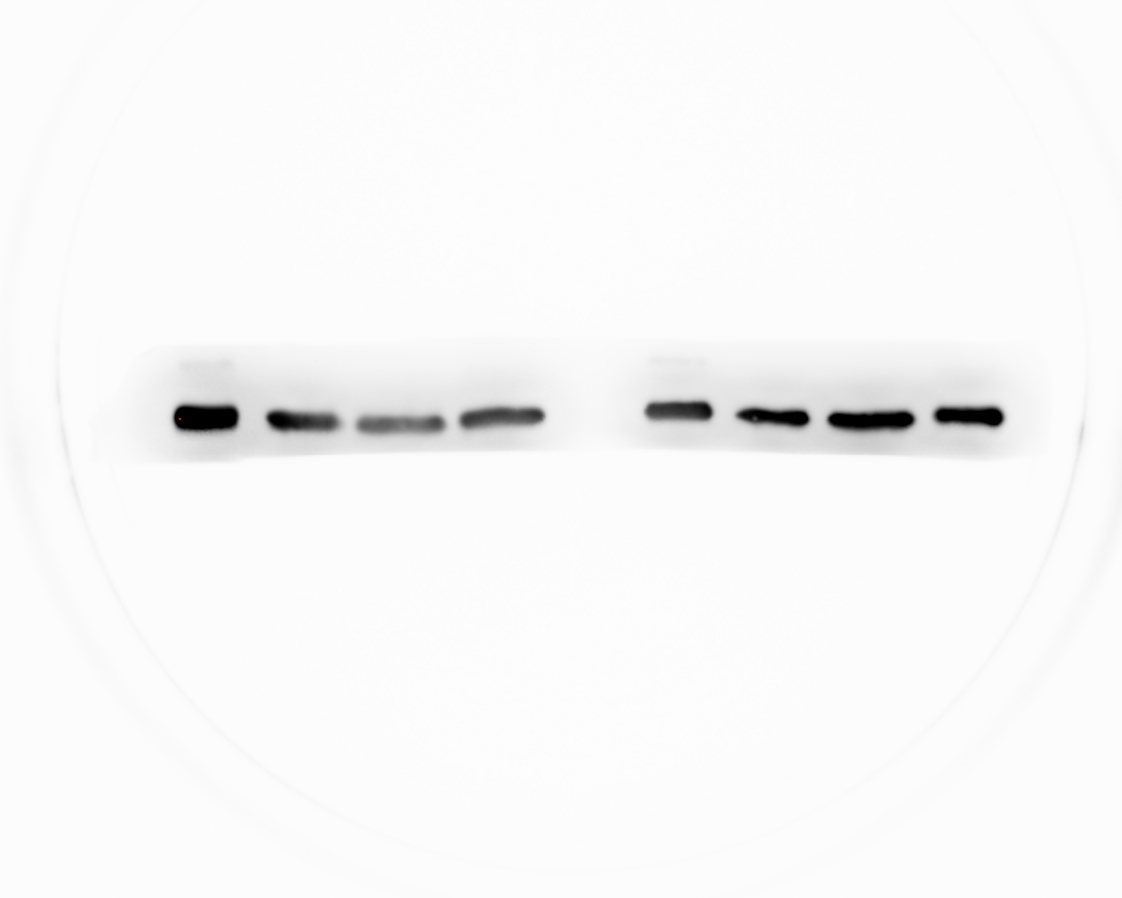

Supplement: Supplementary file 1 — original data files [file 41419_2022_5377_MOESM1_ESM.jpg]

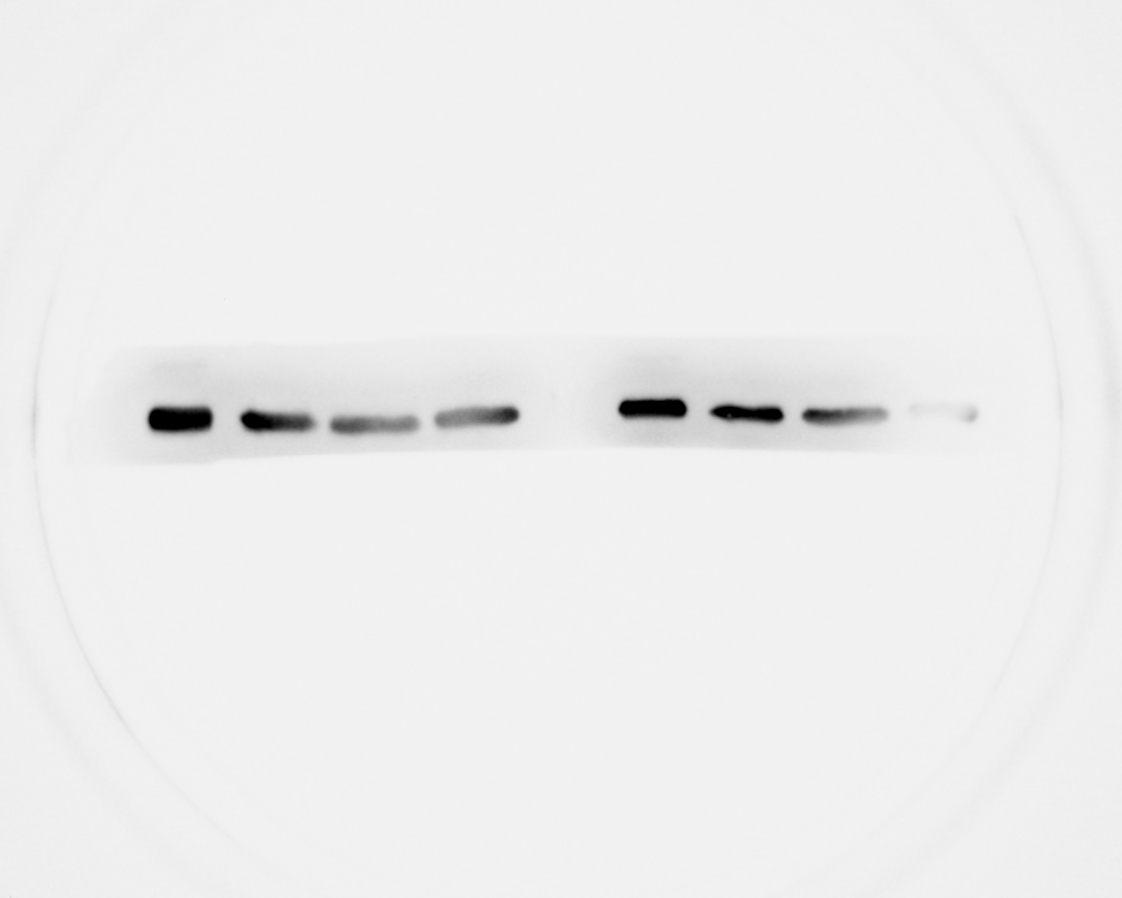

Supplement: Supplementary file 2 — original data files [file 41419_2022_5377_MOESM2_ESM.jpg]

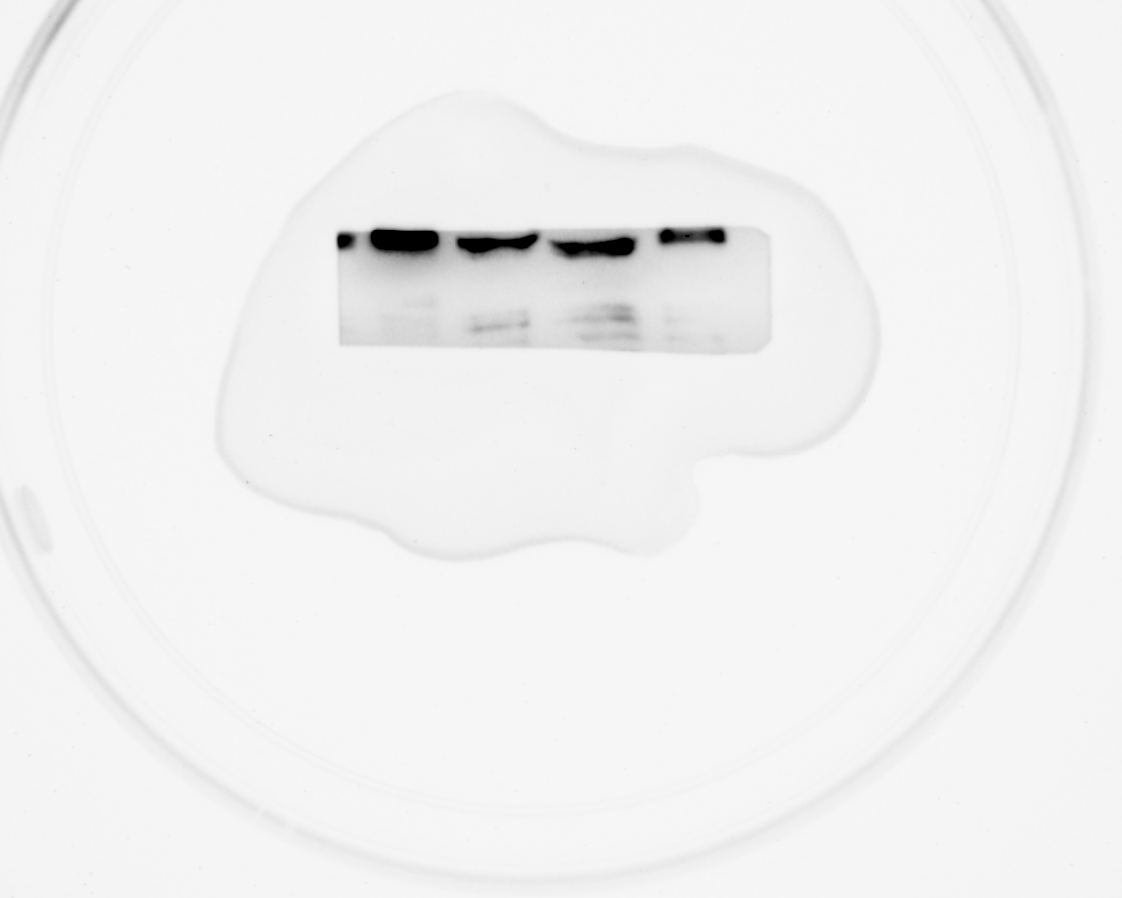

Supplement: Supplementary file 3 — original data files [file 41419_2022_5377_MOESM3_ESM.tif]

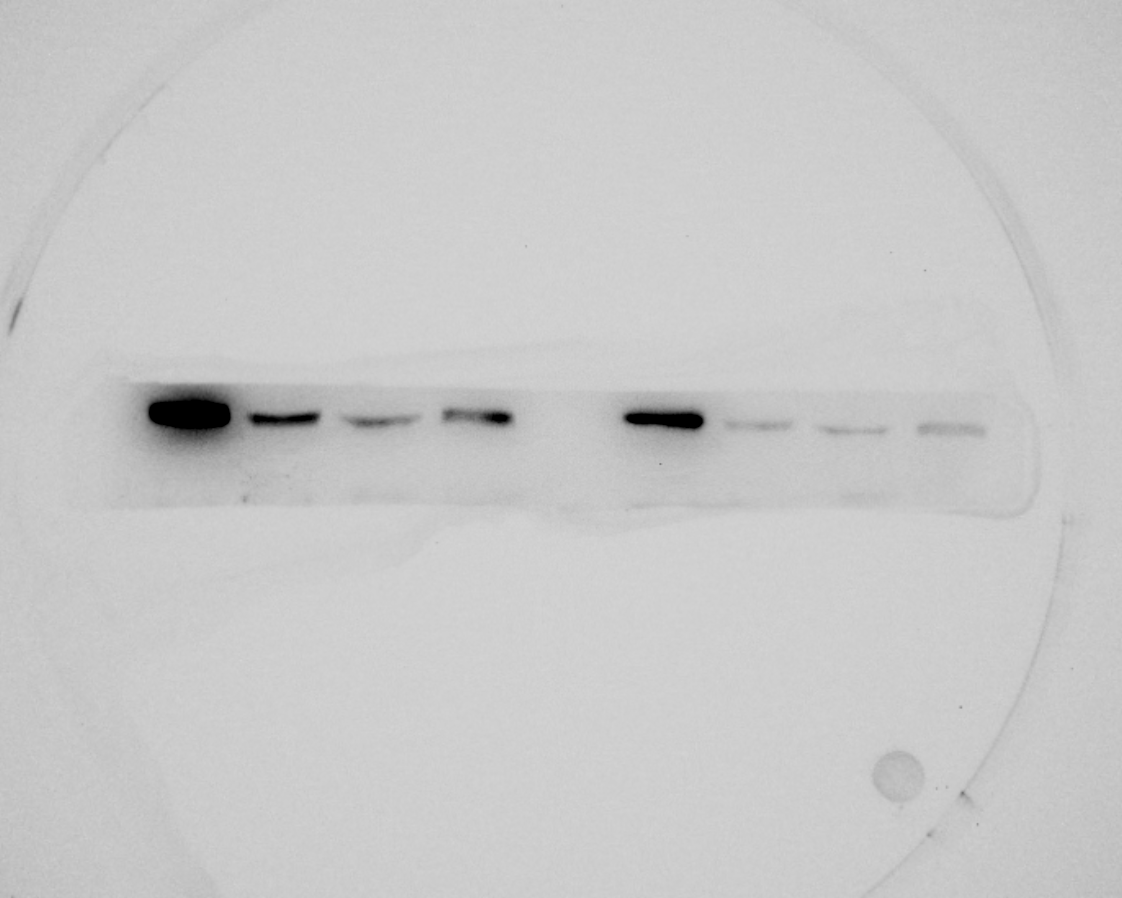

Supplement: Supplementary file 4 — original data files [file 41419_2022_5377_MOESM4_ESM.tif]

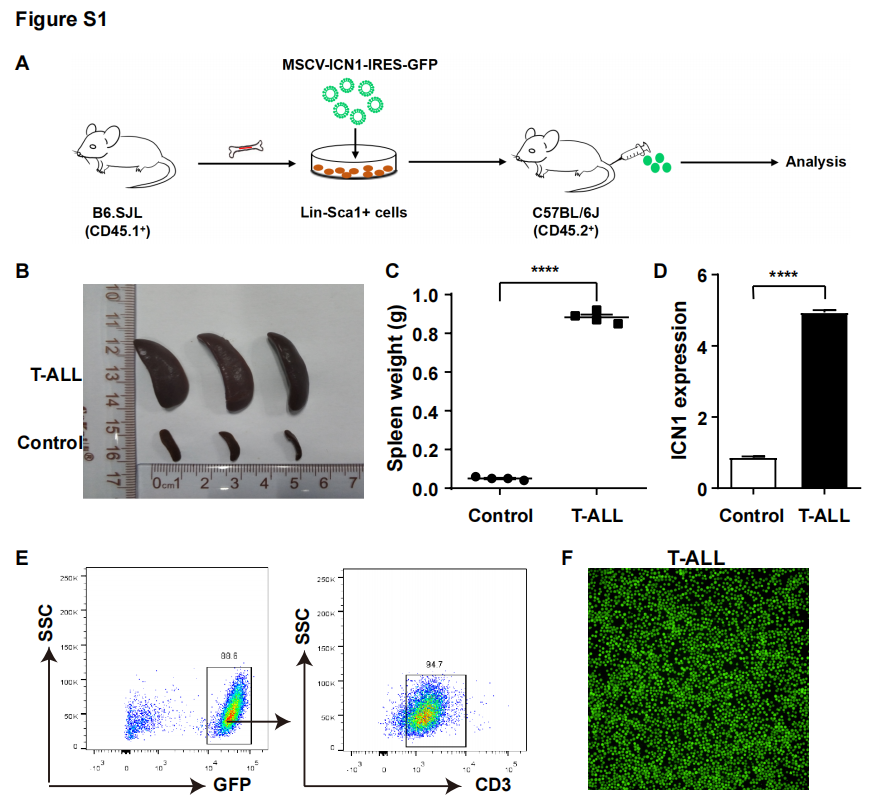

Supplement: Supplementary file 5 — supplemental figure S1 [file 41419_2022_5377_MOESM5_ESM.tif]

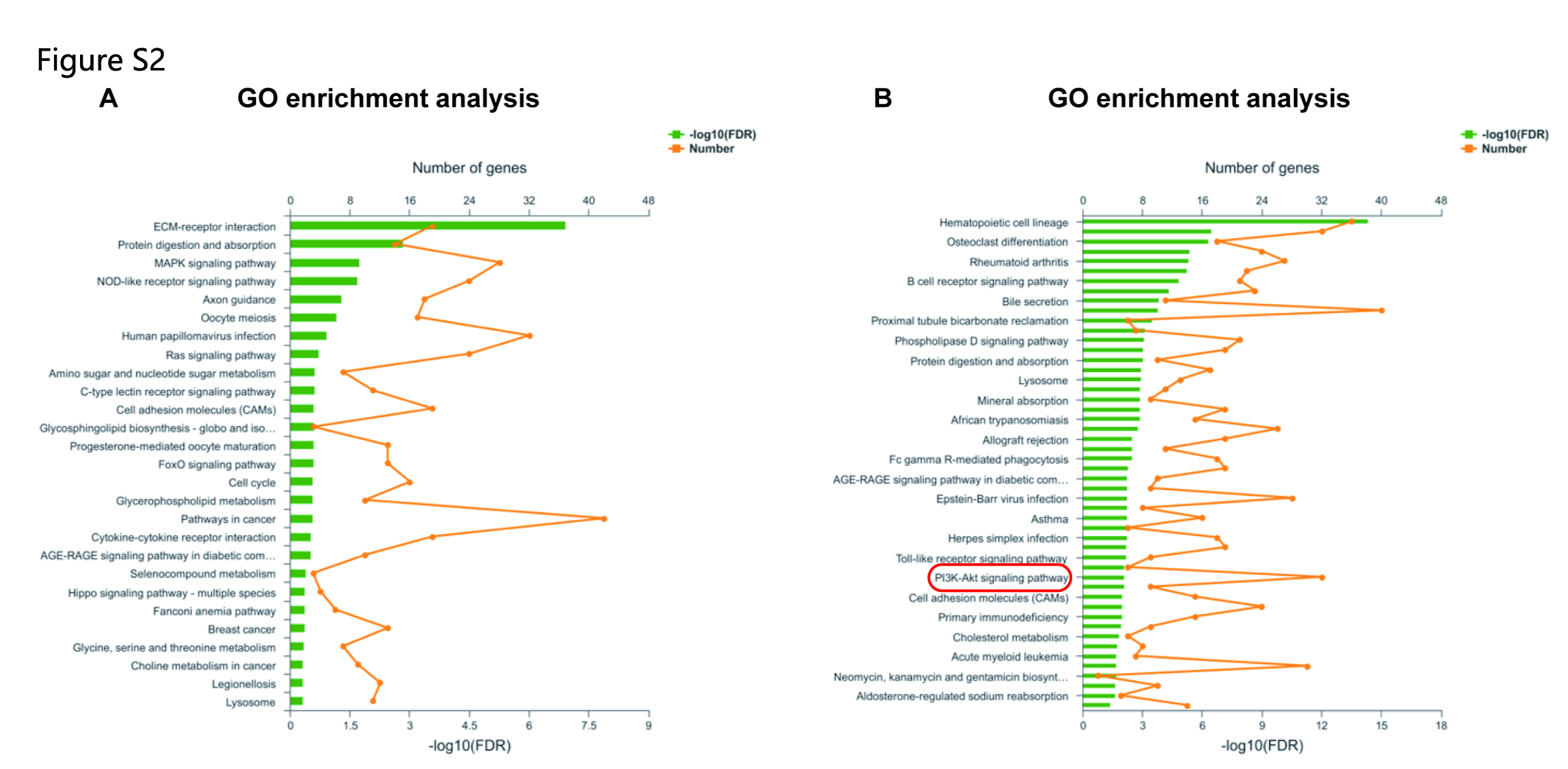

Supplement: Supplementary file 6 — supplemental figure S2 [file 41419_2022_5377_MOESM6_ESM.tif]

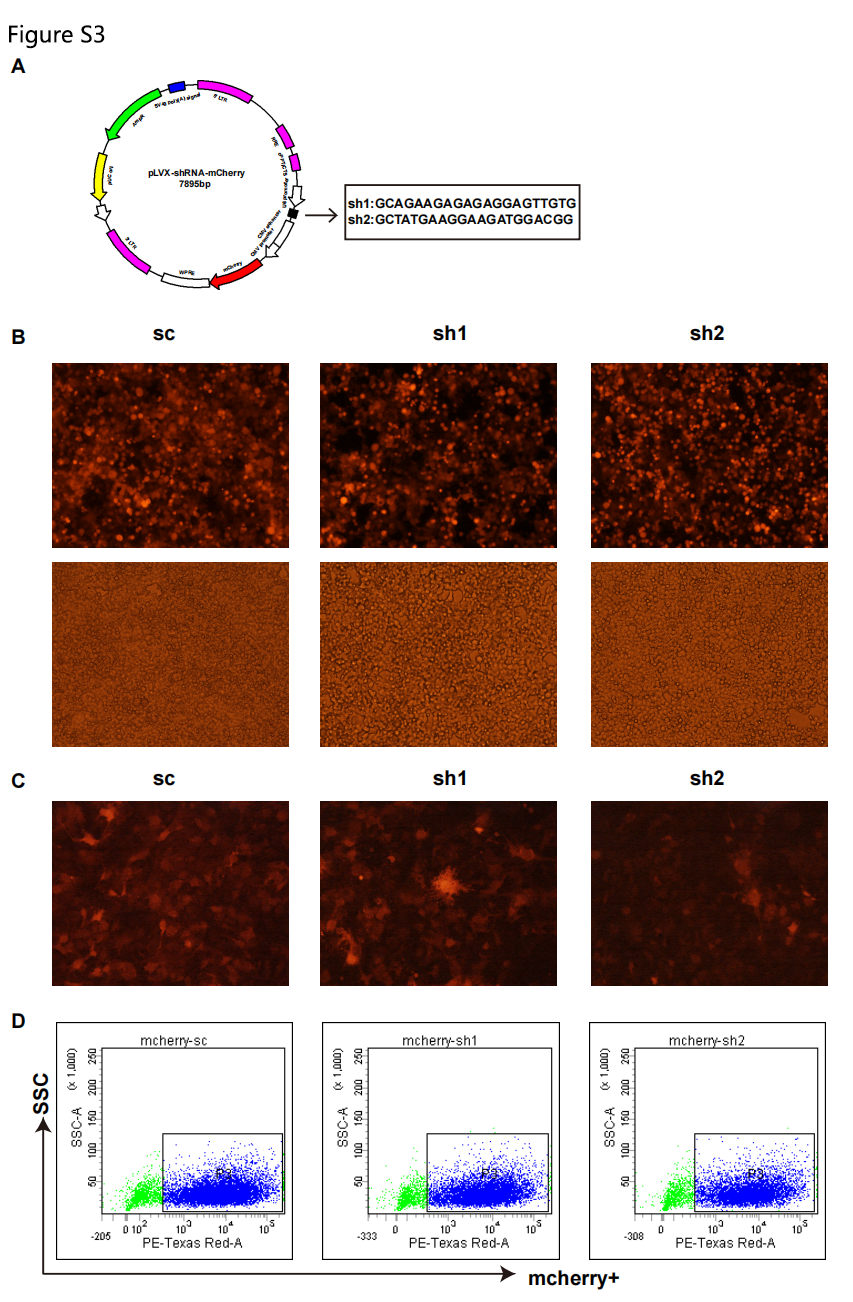

Supplement: Supplementary file 7 — supplemental figure S3 [file 41419_2022_5377_MOESM7_ESM.tif]

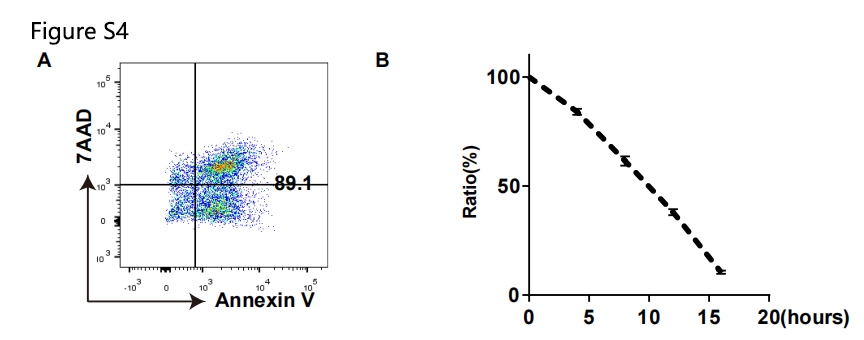

Supplement: Supplementary file 8 — supplemental figure S4 [file 41419_2022_5377_MOESM8_ESM.tif]

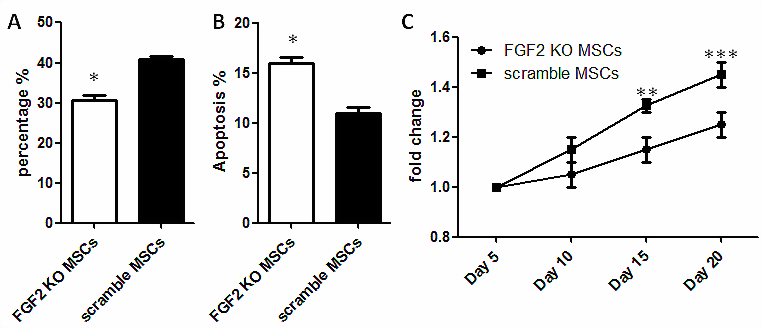

Supplement: Supplementary file 9 — supplemental figure S5 [file 41419_2022_5377_MOESM9_ESM.tif]
