# Supplementary material for: Blockade of FGF2/FGFR2 partially overcomes bone marrow mesenchymal stromal cells mediated progression of T-cell acute lymphoblastic leukaemia
Source: Cell Death Dis. 2022 Nov 4;13(11):922. doi: 10.1038/s41419-022-05377-5 (PMC9636388; doi:10.1038/s41419-022-05377-5)
Supplement: Supplementary file 10 — supplemental figure legend [file 41419_2022_5377_MOESM10_ESM.docx]

**Supplemental Figure Legend**

**Supplemental Figure 1 (Figure S1). Verification of ICN1 overexpression induced T-ALL model.**

1. Establishment of a murine leukemia model by Notch1 overexpression. (B) The spleen size of T-ALL mice was observed on day14 after establishment. (C) The weight of spleen was elevated in T-ALL mice. (D) Relative mRNA expression of ICN1 in leukaemia cells. (E) FACS detected the expression of CD3 in leukaemia cells. (F) Fluorescence images showed that over 90% BM cells were GFP positive.

**Supplemental Figure 2 (Figure S2). GO enrichment analysis of target genes.**

(A) GO enrichment analysis of functions of target genes and signaling pathway of MSCs isolated from BM of T-ALL and control mice. (B) GO enrichment analysis of functions of target genes and signaling pathway of primary murine T-ALL cells co-cultured in vitro with MSCs from T-ALL or control mice.

**Supplemental Figure 3 (Figure S3). Construction of lentivirus-based sh-FGF2 vectors.**

(A) Schematic diagram of lentiviral interference vectors of PLVX-shFGF2-mcherry. (B) Fluorescence (upper) and bright-field (lower) image of 293T cells packaging scramble and sh-RNA. (C) Primary MSCs from T-ALL mice transfected with virus. (D) The expression of mCherry was detected by flow cytometry to verify the transfection efficiency of MSCs.

**Supplemental Figure 4 (Figure S4). Primary murine T-ALL cells die rapidly *in vitro*.**

(A) Apoptosis rate of primary murine T-ALL cells cultured in vitro alone for 16 hours. (B) Growth curve showed that primary T-ALL cells went through apoptosis rapidly *in vitro*.

**Supplemental Figure 5 (Figure S5). FGF2 knockdown attenuated the growth capacity of MSCs.**

MS-5 cells were transfected with FGF2-shRNA and scramble control. (A) The proliferation rate of MS-5 decreased after FGF2 knockdown. (B) The apoptosis rate of MS-5 increased after FGF2 knockdown. (C) Growth curve revealed that the growth capacity of MS-5 decreased after FGF2 knockdown.
