# Supplementary material for: Blockade of FGF2/FGFR2 partially overcomes bone marrow mesenchymal stromal cells mediated progression of T-cell acute lymphoblastic leukaemia
Source: Cell Death Dis. 2022 Nov 4;13(11):922. doi: 10.1038/s41419-022-05377-5 (PMC9636388; doi:10.1038/s41419-022-05377-5)
Supplement: Supplementary file 12 — supplemental table 1 [file 41419_2022_5377_MOESM12_ESM.docx]

| **Antibody** | **Conjugate** | **Clone** | **Source** |
| --- | --- | --- | --- |
| Anti-mouse TER-119 | FITC | TER-119 | Biolegend |
| Anti-mouse CD45 | FITC | 30-F11 | Biolegend |
| Anti-mouse CD31 | FITC | 390 | Biolegend |
| Anti-mouse CD44 | FITC | IM7 | Biolegend |
| Anti-mouse Ly-6A/E (Sca-1) | APC | D7 | Biolegend |
| Anti-mouse CD51 | PE | RMV-7 | Biolegend |
| Anti-mouse c-Kit | PeCy7 | 2B8 | Biolegend |
| Anti-mouse Ly-6A/E (Sca-1) | PeCy7 | D7 | Biolegend |
| Rat IgG2a,κIsotype Ctrl | FITC | RTK2758 | Biolegend |
| Rat IgG2b,κIsotype Ctrl | FITC | RTK4530 | Biolegend |
| Mouse Lineage Isotype Antibody | Pacific Blue |  | BD Biosciences |
| Mouse Lineage Antibody | Pacific Blue |  | BD Biosciences |

**Table S1 Antibodies used for FACS in the study**
