# Supplementary material for: Blockade of FGF2/FGFR2 partially overcomes bone marrow mesenchymal stromal cells mediated progression of T-cell acute lymphoblastic leukaemia
Source: Cell Death Dis. 2022 Nov 4;13(11):922. doi: 10.1038/s41419-022-05377-5 (PMC9636388; doi:10.1038/s41419-022-05377-5)
Supplement: Supplementary file 13 — supplemental table 2 [file 41419_2022_5377_MOESM13_ESM.docx]

**Table S2 Sequences of the primers used for PCR**

| **mRNA** | **Primer sequences** |
| --- | --- |
| Nestin | Forward: GCAGAGAAGACAGTGAGGCAGATG |
|  | Reverse: GGAGGCAGGAGACTTCAGGTAGAG |
| Rux2 | Forward: TGTCCGCCACCACTCACTACC |
|  | Reverse: GAACTGATAGGATGCTGACGAAGTACC |
| Col1a1 | Forward: GACAGGCGAACAAGGTGACAGAG |
|  | Reverse: CAGGAGAACCAGGAGAACCAGGAG |
| Sox9 | Forward: ATGACCGACGAGCAGGAGAAGG |
|  | Reverse: CCGAGGGACAGGGCGAACC |
| VE-cadherin | Forward: CCCACTATCCGATACGAATACC |
|  | Reverse: ATCCACATCTAGGACGTTGATG |
| Pecam-1 | Forward: CACAACAAACAAGCTAGCAAGA |
|  | Reverse:TTTGGCTGCAACTATTAAGGTG |
| Angpt1 | Forward: CGGGAAGATGGAAGCCTGGATTTC |
|  | Reverse: ATGTACTGCCTCTGACTGGTTATTGC |
| Opn/Spp1 | Forward: GACGATGATGATGACGATGGAGACC |
|  | Reverse: CTGTAGGGACGATTGGAGTGAAAGTG |
| GAPDH | Forward: TGAAGGTCGGTGTGAACGGATT |
|  | Reverse:CTCGCTCCTGGAAGATGGTGAT |
| Csf2 | Forward:TTCAAGAAGCTAACATGTGTGC |
|  | Reverse:GGTAACTTGTGTTTCACAGTCC |
| Fgf2 | Forward:AGTTGTGTCTATCAAGGGAGTG |
|  | Reverse:CATTGGAAGAAACAGTATGGCC |
| Igf2 | Forward:GTTGGTGCTTCTCATCTCTTTG |
|  | Reverse:AAACTGAAGCGTGTCAACAAG |
| Ctf1 | Forward:CTCATTCCTACCCCATTTGGAG |
|  | Reverse:ACGTATTCCTCCAGAAGTTGTT |
| Hgf | Forward:ACCTACAGGAAAACTACTGTCG |
|  | Reverse:TGCATTCAACTTCTGAACACTG |
| Lif | Forward:GCTACTATAGACGTCATGAGGG |
|  | Reverse:CAACCCAACTTTTTCCTTTGGA |
| Fgfr1 | Forward:TGGAGTTAATACCACCGACAAA |
|  | Reverse:GATGATGATCTCCAGGTACAGG |
| Fgfr2 | Forward:CTAAAGGCAACCTCCGAGAATA |
|  | Reverse:ACATTTTTGGGAAGCCAAGTAC |
| Fgfr3 | Forward:GAGGACAACGTGATGAAGATCG |
|  | Reverse:GGTTGGTCGTCTTCTTGTAGTA |
| Fgfr4 | Forward:GCGGCGTCCACCACATTG |
|  | Reverse:GTTGTCTTTGAGCATCTTGACG |
| P21 | Forward:CCTTGTCGCTGTCTTGCACTCTG |
|  | Reverse:GCTGGTCTGCCTCCGTTTTCG |
| P27 | Forward:GGGATATGGAAGAAGCGAGTCAGC |
|  | Reverse:TCTCCACCTCCTGCCATTCGTATC |
| Cdk2 | Forward:GAACTTACACTCATGAGGTGGT |
|  | Reverse:GGAAGAGTTGGTCAATCTCAGA |
| Ccnd1 | Forward:TGGATGCTGGAGGTCTGTGAGG |
|  | Reverse:GCAGGCGGCTCTTCTTCAAGG |
| Caspase3 | Forward:GAAACTCTTCATCATTCAGGCC |
|  | Reverse:GCGAGTGAGAATGTGCATAAAT |
| Bax | Forward:TTGCCCTCTTCTACTTTGCTAG |
|  | Reverse:CCATGATGGTTCTGATCAGCTC |
| Bcl-2 | Forward:GATGACTTCTCTCGTCGCTAC |
|  | Reverse:GAACTCAAAGAAGGCCACAATC |
